# Towards Restoring Dopaminergic Balance in Fibromyalgia: The Untapped Potential of Nonpharmacological Interventions

**DOI:** 10.1155/prm/1462017

**Published:** 2026-10-20

**Authors:** Davide Spinetti, Michela Sarlo, Paolo Sfriso, Roberta Ramonda, Sara Bindoli, Marta Ghisi, Daniela Mapelli, Giuseppe Sergi, Marina De Rui, Maria Devita

**Affiliations:** ^1^ Department of General Psychology (DPG) University of Padua Padua Italy unipd.it; ^2^ Department of Communication Sciences, Humanities and International Studies University of Urbino Carlo Bo Urbino Italy uniurb.it; ^3^ Department of Medicine (DIMED) Rheumatology Unit University of Padua Padua Italy unipd.it; ^4^ Department of Surgery, Oncology and Gastroenterology-DISCOG University of Padua Padua Italy unipd.it; ^5^ Unità Operativa Complessa (U.O.C.) Hospital Psychology University-Hospital of Padova Padua Italy; ^6^ Department of Medicine (DIMED) Geriatrics Unit University of Padua Padua Italy unipd.it; ^7^ Geriatric Division Azienda Ospedale Università Padova Padua Italy

**Keywords:** chronic pain, fibro-fog, noninvasive brain stimulation, precision medicine, reward system

## Abstract

Fibromyalgia (FM) is a chronic syndrome characterized by widespread pain, cognitive dysfunction, and mood disturbances. Growing evidence suggests that dopaminergic dysregulation may play a role in the pathophysiology of FM, linking reduced dopamine function to altered pain perception, anhedonia, fatigue, and impaired cognition. While pharmacological treatments—such as duloxetine, milnacipran, and pregabalin—may influence dopamine only indirectly, dopaminergic agonists like pramipexole have shown partial efficacy. Nonpharmacological interventions, including exercise, mindfulness, cognitive behavioral therapy (CBT), and noninvasive brain stimulation (NIBS), consistently improve symptoms, but their potential impact on dopamine pathways remains largely unexplored. This narrative‐short review highlights a critical knowledge gap regarding dopaminergic modulation by nonpharmacological strategies and proposes a paradigm shift to be tested in future FM research. Future longitudinal, multimodal studies integrating neuroimaging, dopaminergic biomarkers, and clinical outcomes are required to test whether combined interventions exert synergistic effects on dopamine circuits. The validation of dopaminergic biomarkers to stratify patients according to their individual neurobiological profiles represents a critical step toward transitioning from a ‘one‐size‐fits‐all’ approach to a precision medicine paradigm, enabling the development of tailored interventions aimed at restoring dopaminergic balance and, ultimately, improving pain, mood, cognition, and quality of life in FM patients.

## 1. Introduction

Fibromyalgia (FM) is a chronic rheumatologic syndrome characterized by widespread musculoskeletal pain and other debilitating systemic symptoms. Among these, cognitive deficits, commonly referred to as *fibro-fog*, together with persistent pain, are consistently described as the most impactful features of FM on daily functioning [1, 2]. The International Association for the Study of Pain (IASP) defines pain as “an unpleasant sensory and emotional experience associated with, or resembling that associated with, actual or potential tissue damage” [3]. When it lasts more than 3 months, it is considered *chronic pain* [4]. Current pharmacological strategies for chronic pain present well‐recognized limitations: addiction, modest clinical efficacy, and weakness in adequately addressing cognitive disturbances [5–7]. Although the neurobiological underpinnings of chronic pain remain incompletely understood, converging evidence suggests a key role for the dopaminergic system. Dopamine has been implicated in pain modulation, reward processing, and a broad range of cognitive functions, including attention, executive function, working memory, processing speed, and both short‐ and long‐term memory [8–12], and alterations in dopamine signaling have been repeatedly documented in chronic pain conditions, such as FM [13–15]. Indeed, Albrecht et al. [5] showed that, compared to healthy controls, FM patients display reduced cortical dopamine D2/D3 receptor binding. Reduced dopaminergic tone has been specifically detected in the anterior cingulate cortex (ACC), a central hub for descending pain modulation and emotional appraisal of pain, as well as in other pain‐ and stress‐related regions, including the parahippocampal gyrus, temporal pole, and hippocampus [16–19]. Dysregulation of mesencephalic dopaminergic nucleus output, namely the Ventral Tegmental Area (VTA), has been linked not only to depression and addiction but also to cognitive dysfunction, further reinforcing the centrality of dopaminergic imbalance in FM [20]. Among pharmacological options, dopaminergic agonists such as pramipexole have shown promise in improving both pain and cognition in FM patients [21–24]. Pramipexole acts on D2 receptors and reduces N‐methyl‐D‐aspartate‐mediated nociception through tyrosine kinase activation, whereas D3 receptor stimulation may normalize mesolimbic arousal abnormalities, contributing to analgesic and cognitive benefits. Similarly, opioids modulate medial prefrontal cortex (PFC) activity in FM, although these results require cautious interpretation [25]. Overall, while multiple pharmacological interventions have been studied in relation to dopaminergic signaling [26, 27], the impact of nonpharmacological strategies on this system remains largely unexplored. To date, only a few studies have indirectly addressed this question. Nonpharmacological interventions, such as physical exercise [28], cognitive behavioral therapy (CBT) [29], psychoeducation, sleep hygiene, yoga, and tai chi [30], have been shown to improve pain, fatigue, mood, and cognition, with some of these effects sometimes corroborated by functional magnetic resonance imaging (fMRI) findings. However, these studies did not assess dopaminergic neurotransmission directly. A notable exception is the FIBRODOPA trial [31], which is investigating whether the mindfulness‐based intervention *Mindfulness-Oriented Recovery Enhancement (MORE)* can restore dopaminergic functioning in FM patients, with potential benefits for reward responsiveness, pain regulation, and mood. Results of this randomized controlled trial, however, have not yet been published. This limitation leaves a critical question unanswered: what long‐lasting neurobiological adaptations are associated with these treatments? Considering that nonpharmacological interventions have been shown to alleviate several FM‐related symptoms closely linked to dopamine signaling, such as fatigue, cognitive dysfunction, and motivational deficits, the absence of research on dopaminergic modulation is noteworthy. Addressing this gap may open novel research avenues. Future work should therefore examine whether dopaminergic mechanisms contribute to the therapeutic effects of these interventions. Highlighting this relationship may help to shed light on pharmacological and nonpharmacological combined treatments for optimizing dopaminergic function and clinical outcomes. The aim of this narrative‐short review is to highlight the critical gap regarding the limited evidence on the effects provided by nonpharmacological interventions on the clinical symptoms usually present in FM and to propose new point of research directions. This may help to shed light on the importance of dopaminergic imbalance in FM.

## 2. Literature Search

The literature considered in this narrative review was identified through searches of the PubMed database. The search covered studies published up to 2025 and focused on the role of dopaminergic mechanisms in FM, pain processing, cognitive and affective symptoms, and the potential effects of pharmacological and nonpharmacological interventions on dopaminergic pathways. Additional relevant articles were identified by screening the reference lists of selected publications. The following keywords were used in different combinations: “fibromyalgia,” “dopamine,” “dopaminergic system,” “dopamine receptors,” “pain,” “cognition,” “mood,” “reward,” “nonpharmacological interventions,” “exercise,” “cognitive behavioral therapy,” “mindfulness,” “transcranial magnetic stimulation,” and “transcranial direct current stimulation.”

### 2.1. Dopamine and FM: A Detrimental or a Beneficial Relationship?

The knowledge of the effects provided by nonpharmacological interventions on dopamine functioning is scarce, prompting questions about their potential long‐term impact on the dopamine system. Neurobiologically, dopamine is synthesized from tyrosine: first hydroxylated to L‐3,4‐dihydroxyphenylalanine by tyrosine hydroxylase, then decarboxylated to dopamine by aromatic L‐amino acid decarboxylase, both tightly regulated rate‐limiting steps [32]. Dopamine acts via five receptors (D1‐D5), broadly grouped as D1‐like (D1/D5, enriched in the PFC, associated with working memory and executive function) and D2‐like (D2/D3/D4, prominent in the striatum, involved in reward and motivational processes), which can guide biomarker selection, such as D2/D3 positron emission tomography (PET) ligands or fluorodopa (FDOPA) for synthesis [5, 10, 13, 20]. D2 receptors, widely distributed across several brain areas, are strongly associated with chronic pain [20] and are implicated in motor control, motivation, reward, sleep, mood, and cognition, including attention, working memory, and learning [13, 20]. In acute pain, nociceptive stimuli activate ascending and descending pathways [33], with the mesolimbic system involved in reward, motivation, and analgesia [34–36]. Dopamine, in fact, plays an antinociceptive role, as shown in the medial thalamus and ACC [37–39]. Chronic pain disrupts dopamine homeostasis, contributing to the transition from subacute to chronic pain [40]. Reduced dopamine activity in chronic pain has been linked to anhedonia, impaired pain modulation, fatigue, and mood disorders. This framework is captured by the concepts of “reward deficiency” and “antireward” [35, 36, 41–43]. This framework is highly relevant to FM, a condition in which widespread chronic pain is the hallmark symptom and in which dopamine dysfunction has been consistently reported [15]. Studies using PET show reduced central nervous system dopamine uptake correlating with heightened pain perception [14]. Stress may drive dopamine reduction in the VTA, amplifying nociception [44–47]. Furthermore, FM patients show an abnormal pain response and decreased dopamine release compared to healthy controls during painful stimulation [5, 9, 36]. Structural and functional alterations in reward pathways have also been reported, including the nucleus accumbens, VTA, and limbic cortex [36, 41, 48]. Modulating these networks could improve pain, mood, motivation, and cognition in FM. Pharmacologically, levodopa, a dopamine precursor, has shown efficacy in reducing pain in conditions such as Parkinson’s disease, herpes zoster, and diabetic polyneuropathy [49], but its utility in FM remains underexplored. Similarly, D2 receptor antagonists such as antipsychotics have yielded inconsistent results [50–52]. Pharmacological agents approved by the Food and Drug Administration for FM are duloxetine, milnacipran, pregabalin, and cyclobenzaprine. Other options include amitriptyline, gabapentin, and naltrexone [53–56]. Although nonpharmacological interventions, such as physical exercise, mindfulness, and CBT, have demonstrated promising effects on pain, mood, fatigue, and daily functioning in FM, to date, no studies have directly assessed whether their benefits in FM are mediated by modulation of dopaminergic signaling.

### 2.2. Nonpharmacological Interventions in FM: Evidence for Short‐Term Efficacy and Open Questions on Long‐Term Effects

Over the years, research has increasingly considered not only pharmacological treatments for FM symptoms, but also nonpharmacological interventions. However, it is important to underscore the challenge these treatments face due to the limited availability of scientifically proven, effective strategies. Complicating therapeutic decision‐making is the fact that the etiology of FM remains unknown. The most widely accepted hypothesis is central sensitization, which leads to hyperalgesia and allodynia [57]. Nonetheless, other proposed mechanisms—including distress, lack of autonomy, neuroinflammation, and immunological factors—cannot be overlooked [54–58]. This lack of etiological clarity adds complexity to treatment selection. Moreover, the pharmacological agents currently used are not without adverse effects [53–56]. It is therefore unsurprising that nonpharmacological interventions have become first‐line therapies [59]. Initial management, regardless of the chosen approach, often begins with psychoeducation [59]. Other interventions with demonstrated efficacy, especially when combined with drugs, include CBT, mindfulness, and aerobic exercise [57]. These strategies improve not only pain, but also fatigue, sleep disturbances, cognition, and overall quality of life. Importantly, both pharmacological and nonpharmacological therapies must be tailored to the patient’s profile. Integrated approaches, such as the FIBROWALK program, have demonstrated the value of such multimodal treatment [60]. Among complementary strategies, diet has also attracted attention. Vegetarian and vegan diets, rich in vitamins, minerals, fiber, and antioxidants, appear particularly effective in reducing inflammation and oxidative stress [61]. Other emerging approaches—such as virtual reality‐based therapies and hyperbaric oxygen therapy—have yielded encouraging results but require larger, longitudinal trials to confirm their long‐term efficacy. Of the various nonpharmacological strategies, noninvasive brain stimulation (NIBS) currently has the most robust scientific support. Techniques such as transcranial magnetic stimulation (TMS) and transcranial direct current stimulation (tDCS) can modulate neuronal activity and alleviate symptoms like pain and cognitive dysfunction [62]. Their mechanisms involve activating descending pain inhibitory pathways, deactivating the salience network, and modulating functional connectivity through plasticity [62]. However, a striking limitation of the existing literature is the lack of longitudinal designs. Aerobic exercise has been shown, in both human and animal models, to upregulate dopaminergic activity and enhances the reuptake, potentially improving reward sensitivity and motivational drive [63, 64]. Similarly, CBT and mindfulness‐based interventions may indirectly modulate dopaminergic signaling by reducing chronic stress [35, 65], probably normalizing hypothalamic‐pituitary‐adrenal axis activity and reshaping top‐down prefrontal control over limbic and reward‐related regions. NIBS techniques, such as TMS and tDCS, may exert more direct effects on dopaminergic function by modulating cortico‐striatal and cortico‐limbic connectivity, including networks involving the ACC, PFC, and ventral striatum [66]. Through activity‐dependent plasticity, these interventions could influence dopamine release and receptor sensitivity, thereby affecting reward processing, pain modulation, and cognitive control [34]. Most studies report short‐term improvements but fail to clarify how these interventions reshape brain function in the long run. This gap raises an important question: what are the sustained neurobiological changes induced by these interventions? Moreover, an even more neglected issue concerns their potential effect on the dopamine system. Given that nonpharmacological interventions can improve several FM symptoms closely tied to dopaminergic signaling, such as fatigue, cognition, and motivation, it is surprising that their role in modulating dopamine pathways has remained unexplored. Recognizing and addressing this gap could open entirely new research directions. Future studies should investigate whether these interventions exert part of their therapeutic effects through dopaminergic modulation. Demonstrating such a link would not only shed light on their mechanisms of action but could provide the foundation for integrative treatment paradigms that embrace both pharmacological and nonpharmacological tools to restore dopamine balance and optimize patient outcomes.

## 3. Trying to Overcome the Current Gap: Future Directions

Recent evidence suggests that the therapeutic effects observed in FM may not arise from isolated mechanisms but from the interaction between biological modulation and experience‐dependent plasticity. We propose a synergistic model where pharmacological agents provide a stable neurobiological substrate by modulating tonic dopamine levels, while nonpharmacological interventions leverage activity‐dependent plasticity to recalibrate phasic reward‐prediction signals. In this perspective, pharmacological and nonpharmacological interventions may act on complementary levels of the same dopaminergic system. The most common pharmacological substances administered in FM, as cited above, are not able to stimulate dopamine receptors directly. Nevertheless, different studies have shown that these molecules may influence dopamine function mainly through indirect mechanisms [67–74]. In parallel, the combination of pharmacological and nonpharmacological treatments is already part of clinical practice. For example, a network meta‐analysis by Nüesch [75] and colleagues concluded that the combination of pregabalin with multicomponent approaches, such as aerobic exercise and CBT, yielded encouraging results. Despite these promising findings, a critical gap remains: it is still unknown whether and how the dopamine system is directly modulated by nonpharmacological interventions. Given the symptomatic improvements consistently observed, it is plausible that the combination of pharmacological and nonpharmacological therapies does not simply act additively on symptoms but may exert a synergistic effect on dopaminergic pathways, restoring reward processing and motivation, and modulating the functional loop between the ACC and the VTA. This framework hypothesizes that the “drug/molecule” acts neurobiologically on the dopaminergic substrate, while the “nondrug intervention” exploits that induced plasticity to reinforce reward learning, reduce hypervigilance, and enhance emotional regulation. Such an interaction could be described as a potential “dopamine reset,” capable of rebalancing reward prediction error signals, reducing the abnormal salience attributed to pain, and fostering compliance, mood stability, and cognitive resilience. To validate this hypothesis, a paradigm shift in clinical research on FM is urgently needed. Future studies should not limit themselves to assessing improvements in pain, mood, or function. Instead, they should incorporate dopaminergic biomarkers as primary outcomes, such as PET imaging of D2/D3 receptor binding, fMRI reward‐related tasks, and behavioral measures of motivation. This would allow researchers to identify which combinations of treatments most effectively modulate the dopaminergic system and ultimately to predict responders more accurately. A concrete proposal is the design of longitudinal, multimodal studies. A feasible trial would randomize FM patients to three arms/groups: (A) pharmacological arm (standard dopaminergic‐acting drug or standard of care medication), (B) nonpharmacological arm (e.g., structured aerobic exercise + CBT or mindfulness), and (C) combined arm. Assessments at baseline, mid‐treatment (e.g., 8 weeks), end‐of‐treatment (e.g., 16 weeks), and follow‐up (6 months) should include clinical outcomes (pain scales, fatigue, quality of life, sleep assessment), multimodal imaging PET [18F‐FDOPA] and PET D2/D3 if feasible, resting‐state and task fMRI, behavioral and cognitive tasks (probabilistic reward learning, N‐back), and expectancy/placebo measures (pretreatment expectancy questionnaires; conditioning paradigm or sham control for NIBS. Primary endpoints should be (1) change in a prespecified dopaminergic biomarker and (2) clinically meaningful fibro‐fog and pain reduction. Sample size planning could be informed by PET effect sizes reported in previous studies, starting from small pilot samples (e.g., 10–20 participants per arm) and progressively increasing sample size in subsequent trials. Expectancy/placebo effects should be measured explicitly and sham controls should be used where possible to dissociate expectancy‐driven dopamine changes from treatment‐specific effects. The rationale for this design is to distinguish the potential dopaminergic effects of pharmacological and nonpharmacological interventions while also exploring whether their combination may produce complementary or additive effects. The inclusion of baseline, mid‐treatment, and end‐of‐treatment assessments would allow the temporal evolution of dopaminergic and clinical changes to be examined, whereas a longer‐term follow‐up could help determine whether any observed changes are sustained beyond the intervention period. Incorporating the biological and behavioral markers presented in Table 1 provides a comprehensive framework to elucidate target interventions and shared mechanistic pathways.

**TABLE 1:** For selected nonpharmacological and pharmacological interventions, a proposed primary biomarker (when available) and a primary behavioral task are reported to facilitate testing of dopaminergic mediation.

Intervention	Hypothesized dopaminergic pathway	Primary biomarker/measure	Behavioral task	Key references
Exercise	Nigrostriatal/Mesolimbic (VTA ⟶ NAcc/Striatum)	D2/D3 binding (PET), fMRI	Effort‐based reward task	Yang et al., [20]
CBT	Mesocortical‐Mesolimbic‐Striatal	fMRI	*Pain Inventory*, *Pain Catastrophizing Scale*, *FIQ-R*	Aman et al., [29]
NIBS (TMS/tDCS)	Mesocortical and mesolimbic pathways	PET [11C] Raclopride	Multidomain battery	Tremblay et al., [66]
Observational study	Multicircuit synergistic modulation	[(18)F]fallypride (FAL)	Multidomain battery	Albrecht et al., [5]

*Note:* The table is illustrative rather than exhaustive and focuses on interventions for which dopaminergic mechanisms can be meaningfully assessed. FAL, ([18]F) fallypride. NAcc, nucleus accumbens.

Abbreviations: CBT, cognitive behavioral therapy; FIQ‐R, fibromyalgia impact questionnaire revised; fMRI, functional magnetic resonance imaging; NIBS, noninvasive brain stimulation; PET, positron emission tomography; tDCS, transcranial direct current stimulation; TMS, transcranial magnetic stimulation; VTA, ventral tegmental area.

Importantly, the interpretation of dopaminergic biomarkers will require particular caution because alterations in dopaminergic signaling may not be specific to FM. Comorbid depressive disorders may independently influence dopaminergic function, while concomitant medications may also affect dopaminergic pathways or related neurobiological measures. Future studies should therefore systematically characterize depressive symptoms and psychiatric comorbidities and document medication use. Such approaches would help distinguish dopaminergic changes specifically associated with FM and its treatment from effects related to comorbidities or medication exposure. Finally, the potential value of dopaminergic biomarkers should initially be considered primarily in relation to understanding disease mechanisms and treatment response rather than establishing diagnostic utility. The identification of dopaminergic alterations in individuals with FM would not, by itself, demonstrate diagnostic specificity. Determining whether such biomarkers can contribute to FM diagnosis or patient classification would require further validation in adequately powered studies including healthy controls and relevant clinical comparison groups, as well as assessment of their diagnostic accuracy and reproducibility.

To conclude, each arm should be evaluated through both neurobiological markers and clinical outcomes, ensuring a comprehensive understanding of short‐ and long‐term effects. Beyond clarifying mechanisms, such studies could also inform personalized medicine approaches, matching patients with the treatment strategies most likely to restore their dopaminergic balance. Embracing this perspective could transform the way we understand and treat FM. If confirmed, the synergistic modulation of the dopaminergic system by combined interventions would not only advance basic neurobiological knowledge but also open concrete avenues for more effective, personalized, and sustainable therapeutic strategies in FM.

### 3.1. Limitations

This narrative review has several limitations. First, the literature search was conducted using PubMed only and was not designed as a systematic review. Therefore, possible other relevant studies indexed in other databases may not have been captured and included. Second, no formal systematic screening or predefined inclusion and exclusion criteria were applied. For this reason, the selection of literature may be subject to selection bias. Finally, the current and available evidence on dopaminergic mechanisms in FM and their modulation by nonpharmacological interventions remains heterogeneous and still limited.

## 4. Conclusions

Dopamine is traditionally associated with reward processing, yet a growing body of evidence indicates that dopaminergic dysfunction also plays a central role in chronic pain conditions, including FM. In FM, altered dopamine signaling has been linked to key clinical features such as chronic pain, mood disturbances, cognitive dysfunction (“fibro‐fog”), and reduced reward responsiveness, fitting within frameworks such as reward deficiency and antireward. Although current pharmacological treatments may influence dopamine only indirectly and nonpharmacological interventions have demonstrated clinical benefits, it remains largely unknown whether these improvements are mediated by dopaminergic mechanisms or whether they reflect sustained neurobiological changes over time. This gap highlights the need for longitudinal and multimodal research explicitly targeting dopaminergic dynamics in relation to treatment response. Specifically, future studies should clarify which nonpharmacological interventions, alone or in combination with pharmacological strategies, hold the greatest promise for restoring a more balanced reward system and dopaminergic signaling in FM. This may allow not only refined treatment personalization but also support the development of interventions with potentially meaningful effects on pain, cognition, mood, and quality of life. While the hypotheses proposed here are necessarily preliminary and based on indirect evidence, systematically tracking dopamine‐related changes alongside clinical outcomes may help advance a more mechanistic and personalized understanding of FM, ultimately moving the field closer to precision medicine.

## Author Contributions

Writing–original draft: Davide Spinetti, Michela Sarlo, Paolo Sfriso, Roberta Ramonda, Sara Bindoli, Marta Ghisi, Daniela Mapelli, Giuseppe Sergi, Marina De Rui, and Maria Devita.

Writing–review and editing: Davide Spinetti, Michela Sarlo, Paolo Sfriso, Roberta Ramonda, Sara Bindoli, Marta Ghisi, Daniela Mapelli, Giuseppe Sergi, Marina De Rui, and Maria Devita. Visualization: Michela Sarlo, Paolo Sfriso, Roberta Ramonda, Sara Bindoli, Marta Ghisi, Daniela Mapelli, Giuseppe Sergi, Marina De Rui, Maria Devita. Supervision: Daniela Mapelli, Giuseppe Sergi, Marina De Rui, and Maria Devita. Conceptualization: Daniela Mapelli, Giuseppe Sergi, Marina De Rui, and Maria Devita.

## Funding

No funding was received for this manuscript.

## Disclosure

All authors have seen and approved the final version of the manuscript being submitted.

## Conflicts of Interest

The authors declare no conflicts of interest.

## Data Availability

Data sharing is not applicable to this article as no datasets were generated or analyzed during the current study.
